# Financial market predictability with tensor decomposition and links forecast

**DOI:** 10.1007/s41109-017-0028-1

**Published:** 2017-05-05

**Authors:** A. Spelta

**Affiliations:** 1 University of Pavia, Pavia, Italy; 2 Complexity Lab in Economics, Milan, Italy; 3 Department of Economics and Management, San Felice 5, 27100 Pavia, Italy

**Keywords:** Networks, Correlations, Tensor decomposition, Forecast

## Abstract

Inspecting financial markets from a complex network perspective means to extract relationships and interdependencies from stock price time series. Correlation networks have been shown to adequately capture such dependence structures between financial assets. Moreover, researchers have observed modifications in the correlation structure between stock prices in the face of a market turbulence. This happens because financial markets experience sudden regime shifts near phase transitions such as a financial crisis. These abrupt and irregular fluctuations from one state to another lead to an increase of the correlation between the units of the system, lowering the distances between the stocks in a correlation network.

The aim of this paper is to predict such abrupt changes by inferring the forthcoming dynamic of stock prices through the prediction of future distances between them. By introducing a tensor decomposition technique to empirically extract complex relationships from prices’ time series and using them in a portfolio maximization application, this work first illustrates that, near critical transitions, there exit spatial signals such as an increasing spatial correlation. Secondly using this information in a portfolio optimization context it shows the ability of the methodology in forecasting future stock prices through these spatial signals. The results demonstrate that an optimization approach aiming at minimizing the interconnectedness risk of a portfolio by maximizing the signals produced by tensor decomposition induces investment plans superior to simpler strategies. Trivially speaking portfolios made up of strongly connected assets are more vulnerable to shock events than portfolios of low interconnected assets since heavily connected assets, being close to a transition point, carry a significant amount of interconnectedness risk, i.e. tail events propagate more quickly to these assets.

## Introduction and literature review

The 2008 financial crisis has shown that financial markets can be considered as prototypes of complex systems in which the simple, microscopic components consist of highly interdependent stocks and the collective behavior is the complex, hard-to-predict behavior of the market as a whole.

Inspecting financial markets from a complex network perspective indeed means to extract relationships and interdependencies from stock price time series in order to understand the factors causing failures in financial markets.

The application of networks to financial markets was pioneered by Mantegna (1999) who showed correlation networks can adequately capture the dependence structure of financial assets. The work of Mantegna (1999) triggered numerous papers that studied financial markets architectures by means of financial networks (Bonanno et al. (2000), Vandewalle et al. (2001), Bonanno et al. (2003), Onnela et al. (2003b) and Tumminello et al. (2010)).

Similar techniques were also applied to investigate the hierarchy structure of currencies as in Mizuno et al. (2005) and Keskin et al. (2011), the behavior of global stock indices as in Roy and Sarkar (2011) and Nobi et al. (2014) and the systemic risk arising from the international banking flows (Spelta and Araújo 2012; Araújo and Spelta 2014; Giudici and Spelta 2016).

Moreover researchers have observed modifications in the correlation structure between stock prices in the face of a market turbulence, such as an ongoing crisis. Onnela et al. (2003a) and Onnela et al. (2003c) analyzing the behavior of correlation networks surrounding the Black Monday (October 19 1987) stock market crash showed that during this crisis the average distances in the correlation network decreased (i.e. increased correlations), while the mean occupation layer shrank implying a more compact network.

Nobi et al. (2014) pointed out that, for the volatile market phases of the 2000s/2010s, from the perspective of correlation networks, major events get visible when the topology of the respective networks drastically changes. In a nutshell, while during expansion and normal periods financial markets tend toward randomness, in crisis phases their structures are reinforced due to a generalized increase in the level of correlations (Onnela et al. 2002; Araújo and Louçã 2007).

Economically, the topological modification of financial networks that occurs during crisis episodes can be associated with the formation and collapse of speculative bubbles. Those bubbles have been largely considered as the consequence of herding behaviors emerging from to the broken balance between autonomous conducts and peer influence (Preis et al. 2011). During crisis, the effect of exchanging influence with the rest of the environment dominates, producing regime shifts that take place at critical thresholds.

Financial markets indeed, being a paradigm of complex systems (Moon and Lu 2015; Preis et al. 2011), experience sudden regime shifts at the so-called tipping points. Regime shifts are associated with critical transitions between alternative states (Dakos et al. 2008; 2010, Kéfi et al. 2014) where fluctuations are characterized by bumps that create upward and downward trends. These abrupt and irregular fluctuations from one state to another lead to an increase in the correlation between the units of the system, lowering in this way the distances between stocks in a correlation network.

The aim of the paper is to predict such abrupt changes by inferring the forthcoming dynamic of stock prices through the prediction of future distances between them (Spelta 2016). In order to accomplish this purpose, first a minimal model of stocks’ behavior is introduced in order to illustrate how, near critical transitions, a phenomenon called critical slowing down can, in theory, generate spatial signals such as an increasing spatial correlation.

Secondly, the paper introduces a tensor decomposition technique to empirically extract complex relationships from prices’ time series and uses them in a portfolio maximization application. Information on future distances indeed uncovers the interconnectedness risk of assets and could be useful in the portfolio optimization context, since it reveals an underlying mechanics of diversification.

Trivially speaking heavily connected assets, being close to a transition point, carry a significant amount of interconnectedness risk, i.e. tail events propagate more quickly to these assets. Assets exhibiting low connectivity on the other hand are less impacted by tail events. Hence, these assets carry relatively less interconnectedness risk. Considering the above argumentation, portfolios made up of strongly connected assets should be more vulnerable to shock events than portfolios of low interconnected assets.

## The proposal

### A minimum model for stock prices dynamic

Large-scale phenomena that occur in financial markets can be associated, using complex system theory, with regime shifts of the complex system underlying the dynamic of the market. Those large-scale phenomena usually take place at critical thresholds - the so-called tipping points - associated with critical transitions between alternative states (Dakos et al. 2008; 2010, Kéfi et al. 2014).

Predicting such critical transitions and abrupt changes in complex systems is a difficult task but fortunately theoretical works (Scheffer et al. 2009; Dakos et al. 2012) suggest the existence of a generic phenomenon, known in dynamical systems theory as critical slowing down, helpful to indicate the proximity of a critical transition even when the knowledge of the functioning of the systems is insufficient for building predictive models.

This phenomenon occurs in most bifurcation points when the dominant eigenvalue characterizing the rates of change of the system around the equilibrium becomes zero (Scheffer et al. 2009). At these points the system becomes increasingly slow in recovering from small perturbations and the resulting time-series turn out to be highly auto-correlated.

Beside the growing auto-correlations of the state variables of the system, recent works (Kéfi et al. 2014; Dakos et al. 2010) have suggested that the critical slowing down phenomenon might, in theory, generate also spatial signals such as an increasing spatial correlation near transitions. This is due to the fact that the entities composing the system pass from isolated to coordinated behaviors, where a spontaneous order emerges (Moon and Lu 2015; Preis et al. 2011; Dakos et al. 2010). When the intrinsic dynamics of each entity is weakened, the state of a unit will be strongly dependent on that of its neighbors. As a result, units will become more strongly correlated close to the transition.

Suppose for instance that the low of motion of two stocks (${s}_1$ and ${s}_2$) is governed by two components, the first ($f$) represents economic fundamentals behind the stocks’ low of motion, the second part ($H$) on the other hand represents the influence between the stocks due to herding behaviors:

$$ \frac{d{ s}_1}{ d t}= f\left({s}_1,{p}_1, c\right)+ H\left({s}_2-{s}_1\right) $$

$$ \frac{d{ s}_2}{ d t}= f\left({s}_2,{p}_2, c\right)+ H\left({s}_1-{s}_2\right) $$

${p}_i$ is a parameter that defines the heterogeneity between the two stocks and $c$ is the control parameter that drives the system to the transition point.

The Jacobian matrix of this system at the equilibrium $\left({s_1}^{*},{s_2}^{*}\right)$ is:

$$ J=\left[\begin{array}{cc}\hfill {f}^{\prime}\left({s_1}^{*},{p}_1, c\right)- H\hfill & \hfill H\hfill \\ {}\hfill H\hfill & \hfill {f}^{\prime}\left({s_2}^{*},{p}_2, c\right)\hfill \end{array}\right] $$

The eigenvalues can be found as:

$$ {\lambda}_{1,2}=\frac{f^{\prime}\left({s_1}^{*},{p}_1, c\right)+{f}^{\prime}\left({s_2}^{*},{p}_2, c\right)}{2}- H\pm \sqrt{{\left[{f}^{\prime}\left({s_1}^{*},{p}_1, c\right)+{f}^{\prime}\left({s_2}^{*},{p}_2, c\right)\right]}^2+2{H}^2} $$

If the system is far away from the transition, in a “business as usual configuration”, the herding behavior is very low $H\ll {f}^{\prime}\left({s_i}^{*},{p}_i, c\right)$ and the fundamentals will govern the dynamic of the stocks. This renders the eigenvalues of the system equal to:

$$ {\lambda}_1={f}^{\prime}\left({s_1}^{*},{p}_1, c\right) $$

$$ {\lambda}_2={f}^{\prime}\left({s_2}^{*},{p}_2, c\right) $$

implying that the two stocks can be regarded as being disconnected, each stock is governed by its own dynamics. As a consequence, one would expect to find no correlation between stock prices.

Prior to a financial crisis, when the system is close to the transition point, the dynamic of the fundamentals of each stock becomes smaller due to critical slowing down ${f}^{\prime}\left({s_i}^{*},{p}_2,{c}^{*}\;\right)\to 0$. The dynamic of the herding behavior, on the other hand, is independent of the proximity to the transition but depends only on the gradient between the two units thus the eigenvalues of the system approach:

$$ {\lambda}_1=0 $$

$$ {\lambda}_2=2 H $$

The dynamic of a stock will be strongly dependent on that of its neighbor. As a result, stocks will become more strongly correlated close to the transition.

## Tensor decomposition and links forecast

Modeling stock price time series with the aim of forecasting their movements is not an easy task because of the lack of knowledge about the drivers of stocks’ dynamic. On the other hand, the previous model and the extensive literature about financial networks has suggested that the relevance of correlation networks relies on the representation of changes follow on the occurrence of stress events.

The rise of the spatial correlation is indeed an helpful indicator of the proximity of a critical transitions such as a financial crisis, even when the knowledge of the functioning of the systems is insufficient for building predictive models.

The scope of this Section is to propose a tensor decomposition methodology (Dunlavy et al. 2011; Gao et al. 2011) to empirically extract complex relationships from prices’ time series and use them for inferring the forthcoming dynamic of stock prices through the prediction of future distances between them (Spelta 2016).

This issue amounts to a link prediction problem (Lü and Zhou 2011). Given past distances between stocks, what will be their next period value? If predictions suggest a contraction of the next period distances for instance, than, we could expect a decrease in prices because of a strengthening of correlations and a higher likelihood of a crisis episode.

The mainstream classes of link prediction methods, are based on the so-called similarity-based algorithms, which are further classified into three categories: local, global and quasi-local depending on the information used (Lü and Zhou 2011). Usually all these techniques (Lü and Zhou 2011) collapse the temporal data into a single matrix by summing (with or without weights) the records corresponding to the each time period. Then similarity-based measures like the Katz centrality (Katz 1953) or the singular value decomposition (SVD) are applied to perform links prediction.

The approach presented in the paper, on the other hand, prevents the temporal aggregation of the data, avoiding losses of crucial features of the system that can be observed only by holding the original time-varying nature of the records.

Starting from $N$ time series of stock prices, a rolling window of length ${n}_1$ is applied to compute the correlation ${C}_{k, l}$ among each pair $\left( k, l\right)$ of instruments. Given these pair-wise correlations, at each time step, a distance matrix with elements:

1 $$ {d}_{k l}=\sqrt{2\left(1-{C}_{k, l}\right)} $$

is created (Mantegna 1999). The financial network is represented by the set of $N$ stocks linked by edges that denote the pair-wise Euclidian distances between those objects. In so doing each link codifies the similarity between the time series of the stocks incident on that link (Javarone and Armano 2013).

Once the rolling window has produced $Z$ distance matrices $\boldsymbol{D}\in {\mathbb{R}}^{N\times N}$, these matrices are embedded into a 3D-tensor $\mathcal{D}\in {\mathbb{R}}^{N\times N\times Z}$ whose generic element ${\delta}_{klz}$ represents the distance between stock $k$ and stock $l$ at time $z$.

The tensor $\mathcal{D}$ is thus approximated as the outer product of three vectors thought the canonical decomposition (Carroll and Chang 1970), also known as parallel factorization (Harshman 1970), the so-called CP decomposition. This technique can be regarded as a generalization of SVD to tensors.

The decomposition aims at writing the $\mathcal{D}$ as the outer product of two identical vectors **v**, that contain the *total spatial dissimilarity* between stocks and a vector **u**, containing the *temporal profile* of the dissimilarities:

2 $$ \mathcal{D}\cong \beta \mathbf{v}\circ \mathbf{v}\circ \mathbf{u} $$

where the symbol “$\circ$” denotes the outer-product, $\mathbf{v}\in {\mathbb{R}}^N,$ $\mathbf{u}\in {\mathbb{R}}^Z$ and $\beta =\parallel \mathbf{v}\parallel \parallel \mathbf{v}\parallel \parallel \mathbf{u}\parallel$.

For an interpretational viewpoint, while a stock with a high (low) total spatial dissimilarity score has, on average, a different (similar) behavior compared with the one of the rest of the market, a period in which a high (low) temporal profile score is registered will be a period in which most of the financial instruments are highly dissimilar (similar).

The problem formulated in Equation (2) is equivalent to minimizing the Frobenius norm of the difference between $\mathcal{D}$ and $\beta \mathbf{v}\circ \mathbf{v}\circ$ $\mathbf{u}$. Solving this problem amounts at finding the rank-1 tensors that best approximate $\mathcal{D}$:

3 $$ m i{n}_{\mathbf{v},\mathbf{v},\mathbf{u}}\parallel \mathcal{D}-\beta \mathbf{v}\circ \mathbf{v}\circ \mathbf{u}{\parallel}_F^2 $$

The 3-dimensional problem is divided into 3 sub-problems by unfolding the tensor $\mathcal{D}$. This means reordering the elements of a tensor into a matrix. The mode-3 unfolding of a tensor $\mathcal{D}$ is denoted by ${\boldsymbol{D}}_{(q)}$ and arranges the mode-q fibers to be the columns of the resulting matrix. Tensor elements $\left( k, l, z\right)$ maps to matrix element $\left({i}_Q, j\right)$, where ${i}_Q=\left\{\mathrm{k},\mathrm{l},\mathrm{z}\right\}$ and $j=1+\sum_{r=1, k\ne q}^z\left({i}_r-1\right){J}_r$ with ${J}_r={\prod}_{m=1, m\ne q}^{r-1}{I}_r$.

The three resulting matrices have respectively a size of $N\times N Z$, $N\times N Z$ and *Z* × *N* ^2^. In this way problem (3) is equivalent to minimizing the difference between each of the modes and its respective approximation in terms of factors.

Problem (3) is thus converted into three problems:

4 $$ \begin{array}{l} mi{n}_{\mathbf{v}>0} \parallel {\mathbf{D}}_{(1)}-\beta \mathbf{v}{\left(\mathbf{u}\odot \mathbf{v}\right)}^T{\parallel}_{\boldsymbol{F}}^{\mathbf{2}}\\ {}\  mi{n}_{\mathbf{v}>0} \parallel {\mathbf{D}}_{(2)}-\beta \mathbf{v}{\left(\mathbf{u}\odot \mathbf{v}\right)}^T{\parallel}_{\boldsymbol{F}}^{\mathbf{2}}\\ {} mi{n}_{\mathbf{u}>0} \parallel {\mathbf{D}}_{(3)}-\beta \mathbf{u}{\left(\mathbf{v}\odot \mathbf{v}\right)}^T{\parallel}_{\boldsymbol{F}}^{\mathbf{2}}\end{array} $$

Where “$\odot$” denotes the Khatri-Rao product, namely the column-wise Kronecker product. Since distances are always non negative, a non-negative tensor factorization method is employed to solve (4) because it greatly simplifies the interpretation of the resulting decomposition. The Block Coordinate Descent Method for Regularized Multiconvex Optimization (Xu and Yin 2013) and the Matlab Tensor Toolbox (Bader and Kolda 2012) are used to solve (4).

After having initialized $\mathbf{v}$ and $\mathbf{u}$ as vectors of ones, the $\mathbf{v}$ component is updated according to the following rules. First compute (5), (6) and (7) as:

5 $$ {\boldsymbol{B}}_{\mathbf{v}}^{k-1}=\mathbf{u}\odot \mathbf{v} $$

6 $$ {\boldsymbol{L}}_{\boldsymbol{v}}^{k-1}=\left|{\left({\mathbf{B}}_{\mathbf{v}}^{\mathrm{k}-1}\right)}^{\mathrm{T}}\left({\mathbf{B}}_{\mathbf{v}}^{\mathrm{k}-1}\right)\right| $$

7 $$ {\mathrm{w}}_{\mathbf{v}}^{\mathrm{k}-1}= \min \left(\frac{{\mathrm{t}}_{\mathrm{k}-1}-1}{{\mathrm{t}}_{\mathrm{k}}},\uptheta \sqrt{\frac{{\mathbf{L}}_{\mathbf{v}}^{\mathrm{k}-1}}{{\mathbf{L}}_{\mathbf{v}}^{\mathrm{k}-2}}}\right) $$

where $$ \theta <1 $$, $$ {t}_0=1 $$, $$ {t}_k=\raisebox{1ex}{$1$}\!\left/ \!\raisebox{-1ex}{$2$}\right.\left(1+\sqrt{1+4{t}_{k-1}^2}\right) $$ with $$ k $$ being the updating step. Secondly compute Eq. (8) as:

8 $$ {\widehat{\mathbf{v}}}^{\mathrm{k}-1}={\mathbf{v}}^{\mathrm{k}-1}+{\mathrm{w}}_{\mathbf{v}}^{\mathrm{k}-1}\left({\mathbf{v}}^{\mathrm{k}-1}-{\mathbf{v}}^{\mathrm{k}-1}\right) $$

And the gradient (9) is:

9 $$ {\boldsymbol{G}}_{\boldsymbol{v}}^{k-1}=\left({\widehat{\boldsymbol{v}}}^{k-1}{\left({\mathbf{B}}_{\mathbf{v}}^{\mathrm{k}-1}\right)}^{\mathrm{T}}-{\boldsymbol{D}}_{(2)}\right){\mathbf{B}}_{\mathbf{v}}^{\mathrm{k}-1} $$

The updating rule for $\mathbf{v}$ is:

10 $$ {\mathbf{v}}^{\mathrm{k}}= \max \left(0,{\widehat{\mathbf{v}}}^{\mathrm{k}-1}-{\mathbf{G}}_{\mathbf{v}}^{\mathrm{k}-1}/{\mathbf{L}}_{\mathbf{v}}^{\mathrm{k}-1}\right) $$

Similarly the $\mathbf{u}$ component is updated according to the following rules. First compute:

11 $$ {\mathbf{B}}_{\mathbf{u}}^{\mathrm{k}-1}=\mathbf{v}\odot \mathbf{v} $$

12 $$ {\mathbf{L}}_{\mathbf{u}}^{\mathrm{k}-1}=\left|{\left({\mathbf{B}}_{\mathbf{u}}^{\mathrm{k}-1}\right)}^{\mathrm{T}}\left({\mathbf{B}}_{\mathbf{u}}^{\mathrm{k}-1}\right)\right| $$

13 $$ {\mathrm{w}}_{\mathbf{u}}^{\mathrm{k}-1}= \min \left(\frac{{\mathrm{t}}_{\mathrm{k}-1}-1}{{\mathrm{t}}_{\mathrm{k}}},\uptheta \sqrt{\frac{{\mathbf{L}}_{\mathbf{u}}^{\mathrm{k}-1}}{{\mathbf{L}}_{\mathbf{u}}^{\mathrm{k}-2}}}\right) $$

Secondly let:

14 $$ {\widehat{\mathbf{u}}}^{\mathrm{k}-1}={\mathbf{u}}^{\mathrm{k}-1}+{\mathrm{w}}_{\mathbf{u}}^{\mathrm{k}-1}\left({\mathbf{u}}^{\mathrm{k}-1}-{\mathbf{u}}^{\mathrm{k}-1}\right) $$

The gradient is:

15 $$ {\mathbf{G}}_{\mathbf{u}}^{\mathrm{k}-1}=\left({\widehat{\mathbf{u}}}^{\mathrm{k}-1}{\left({\mathbf{B}}_{\mathbf{u}}^{\mathrm{k}-1}\right)}^{\mathrm{T}}-{\mathbf{D}}_{(3)}\right){\mathbf{B}}_{\mathbf{v}}^{\mathrm{k}-1} $$

The updating rule for $\mathbf{u}$ is:

16 $$ {\mathbf{u}}^{\mathrm{k}}= \max \left(0,{\widehat{\mathbf{u}}}^{\mathrm{k}-1}-{\mathbf{G}}_{\mathbf{u}}^{\mathrm{k}-1}/{\mathbf{L}}_{\mathbf{u}}^{\mathrm{k}-1}\right) $$

Similarly to the TOPHITS algorithm (Kolda and Bader 2006), the total spatial dissimilarity score of a generic stock $i$ is found as a function of the scores of the rest of the financial instruments weighted by the product of the distances connecting them to stock $i$, and of the temporal profile score of the period in which the distances are observed. The temporal profile score attached to a period, on the other hand, is a weighted sum of the distances recorded in that period. Where each distance is weighted by the product of the spatial dissimilarity score of the stocks connected by such distance.

In this way, the spatial dissimilarity vectors retain also elements representing the temporal evolution of the distances and only the “next step” value of the temporal profile vector has to be inferred from past data. This is a perspective not available when computing link predictions using matrix-based approaches.

A temporal link prediction naturally follows from the decomposition and can be used to infer future distances between stocks, and, on the basis of these forecasts, to predict their future movements.

The next step consists in generating the matrix of the forecasted distances $\widehat{\boldsymbol{D}}$. Instead of predicting the ${N}^2$ possible distances using ${N}^2$ data points, with this method one has to predict only the next value of the temporal profile $\mathbf{u}$ and use it, together with the two fixed total spatial dissimilarity vectors **v**, to build the matrix of the forecasted distances.

An exponential smoothing, applied to the last ${n}_2$ observations of the temporal profile vector **u**, extracts a scalar $\tau$ representing the presumed value of this vector in the next period:

17 $$ \tau =\alpha \left[{\mathbf{u}}_z+\left(1-\alpha \right){\mathbf{u}}_{z-1}+\dots +{\left(1-\alpha \right)}^{n_2-1}{\mathbf{u}}_{z-{n}_2-1}\right]+{\left(1-\alpha \right)}^{n_2}{\mathbf{u}}_{z-{n}_2} $$

Then the matrix containing the forecast of the distances between all instrument pairs is obtained as a linear combination of the two spatial dissimilarity vectors **v**, the parameter $\beta$ and of the forecast $\tau$ of the temporal profile vector as in Eq. (18):

18 $$ \widehat{\boldsymbol{D}}=\beta \tau \mathbf{v}{\mathbf{v}}^{\mathrm{T}} $$

Or, element-wise, $\widehat{d_{k l}}=\beta \tau {\mathbf{v}}_k{\mathbf{v}}_l$.

Given the forecasted distance matrix, the connection intensity to each asset with respect to the other assets can be quantified via a centrality score by summing the distances that link each asset to the others:

19 $$ \widehat{F_k}=\frac{1}{N}{\displaystyle \sum l}\widehat{d_{k, l}} $$

When the steps of the moving window exceed the parameter $Z$, the tensor is allowed to move in time as new data become available. The temporal shift of the tensor permits to compare the forecasts produced by two consecutive decompositions. The difference between the values of the two predictions generates a signal whose sign indicates the future direction of the stock prices. The signal for each stock is obtained as the difference between the centrality values obtained in the two subsequent predictions:

20 $$ {\varDelta_k}^{t+1}={\widehat{F_k}}^t-{\widehat{F_k}}^{t-1} $$

**Fig. 1:**
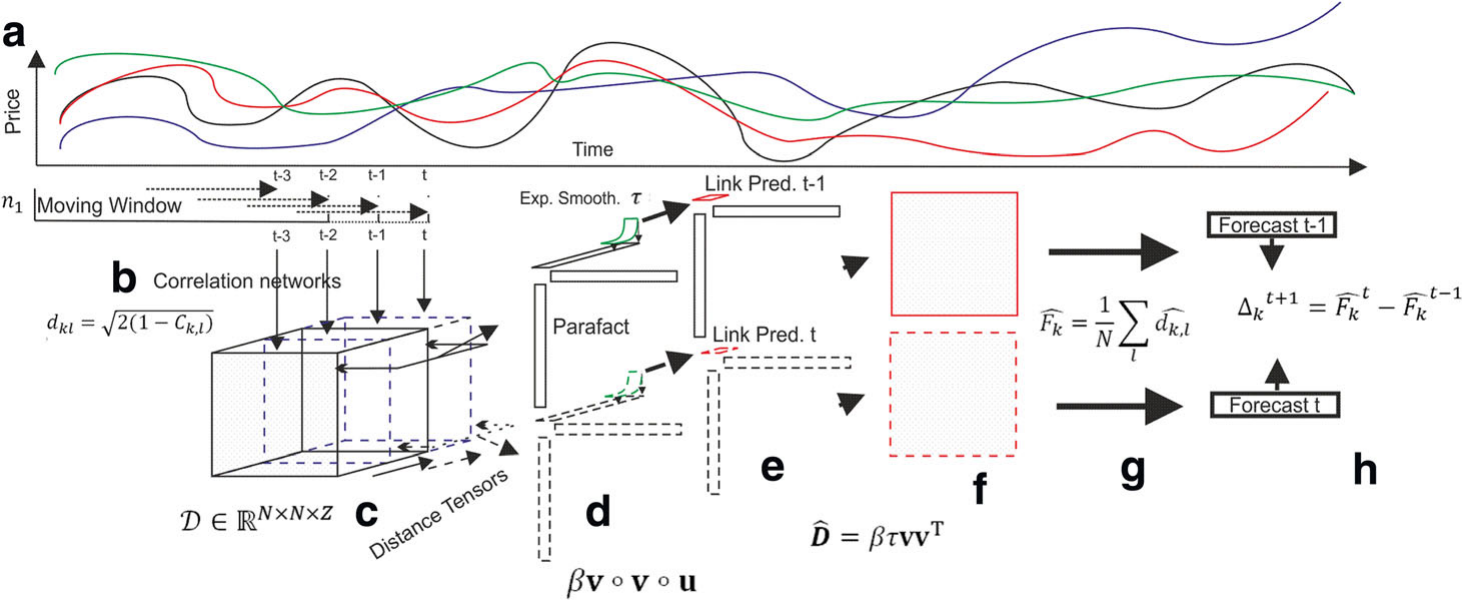
Graphical representation of the method. Starting from stock price time series (**a**), a rolling window is applied to compute the correlation among each pair of stocks (**b**). At each time step a distance matrix is created. Once the rolling window has produced $\mathrm{Z}$ distance matrices, those matrices are embedded into a 3D-tensor (**c**). When the steps of the moving window exceed $\mathrm{Z}$, the tensor is allowed to move in time with each new step, as new data are available (**c** - *solid line* vs. *dashed line*). This temporal shift of the tensor permits to compare the forecasts produced by two consecutive decompositions and generates a signal whose sign indicates the future direction of the stock prices. For graphical purposes, the decomposition of two consecutive tensors and the resulting links prediction are drawn using solid and dashed lines. The two consecutive tensors are approximated as the linear combination of three vectors (**d**) representing spatial ($\mathbf{v}$) and temporal ($\mathbf{u}$) relationships between stocks; $\mathcal{D}\cong \upbeta \mathbf{v}\circ \mathbf{v}\circ \mathbf{u}$. The exponential smoothing (**d** - *green lines*) applied to $\mathbf{u}$ extracts a scalar $\uptau$ representing the forecast of temporal profile for the next period (**e** - *red squares*). The forecast of the future distance matrix is obtained as a linear combination of the two spatial dissimilarity vectors, the parameter $\upbeta$ and of the forecast $\uptau$ of the temporal profile vector; $\widehat{\mathbf{D}}=\uptau \upbeta \mathbf{v}{\mathbf{v}}^{\mathrm{T}}$ (**e** and **f** - *red squares*). Finally, the connection intensity of each asset is quantified via a centrality score by summing the distances that link each asset to the others (**g**). The signal for each stock is obtained as the difference between the centrality values obtained in two predictions (**h**)

To investigate whether this method is able to correctly identify changes in stock prices a back-test based on an hypothetical investment strategy is implemented in the next Section. Figure 1 gives a graphical representation of the technique.

## An investment strategy based on the forecasted distances

This Section aims at creating a dynamic investment strategy based on signal-optimized portfolios. As discussed in the first Section, assets related to negative signals, being close to a transition point, are the more strongly connected and therefore get more easily infected by negative return shocks, leading to subpar risk-adjusted returns.

To avoid this occurrence I apply a portfolio management framework and a risk-based methodology . The optimization approach aims at minimize the interconnectedness risk of the portfolio maximizing the signals produced by the tensor decomposition.

The linear programming optimization is set up as follows:

21 $$ \boldsymbol{\omega} = argma{x}_{\boldsymbol{\omega}}\left[\omega {\boldsymbol{\varDelta}}^T\right] $$

22 $$ s. t.\ {1}^T\boldsymbol{\omega} \le 1 $$

23 $$ 0\le {\omega}_k\le a $$

The variable $\boldsymbol{\varDelta}$ is the $N\times 1$ vector containing the signals, hence the optimization (21) maximizes the weighted portfolio signals. The first constrain (22) implies no leverage, assuming the total wealth to be equal to one. It also indicates that it is not always true that the whole wealth must be allocated in every period. Suppose for instance that a systemic crisis will occur, this will lead all the signals to be negative, and the optimal strategy in this case will be not to trade at all. The second constraint (23) means that weights are subject to lower bound (zero in this case) and upper bound defined by the parameter $a$.

In order to demonstrate the superior performance of the maximized portfolio signals, the investment strategy is compared against an equally weighted portfolio investment methodology where the weights are equal to ${\omega}_k=\frac{1}{N}$. The returns at time $t$ are computed as in Eq. (24):

24 $$ {\mathrm{R}}^{\mathrm{t}}={\displaystyle {\sum}_{\mathrm{k}=1}^{\mathrm{N}}}\left[{\upomega}_{\mathrm{k}}\left(\frac{{{\mathrm{P}}_{\mathrm{k}}}^{\mathrm{t}+1}-{{\mathrm{P}}_{\mathrm{k}}}^{\mathrm{t}}}{{{\mathrm{P}}_{\mathrm{k}}}^{\mathrm{t}}}\right)\right] $$

## Application

The method is applied to three datasets. The first contains 388 stocks belonging to the S&P500 whose closure price changes during 3827 working days, from 1998-01-02 to 2013-08-09. The second dataset regards the closure price of 59 stocks traded over 715 working days, during the period 2014-05-23 to 2017-02-16 in the FTSE MIB while the third sample represents the closure price of 156 stocks traded over the same period of the second dataset but in the Euronext Paris stock exchange.

**Fig. 2:**
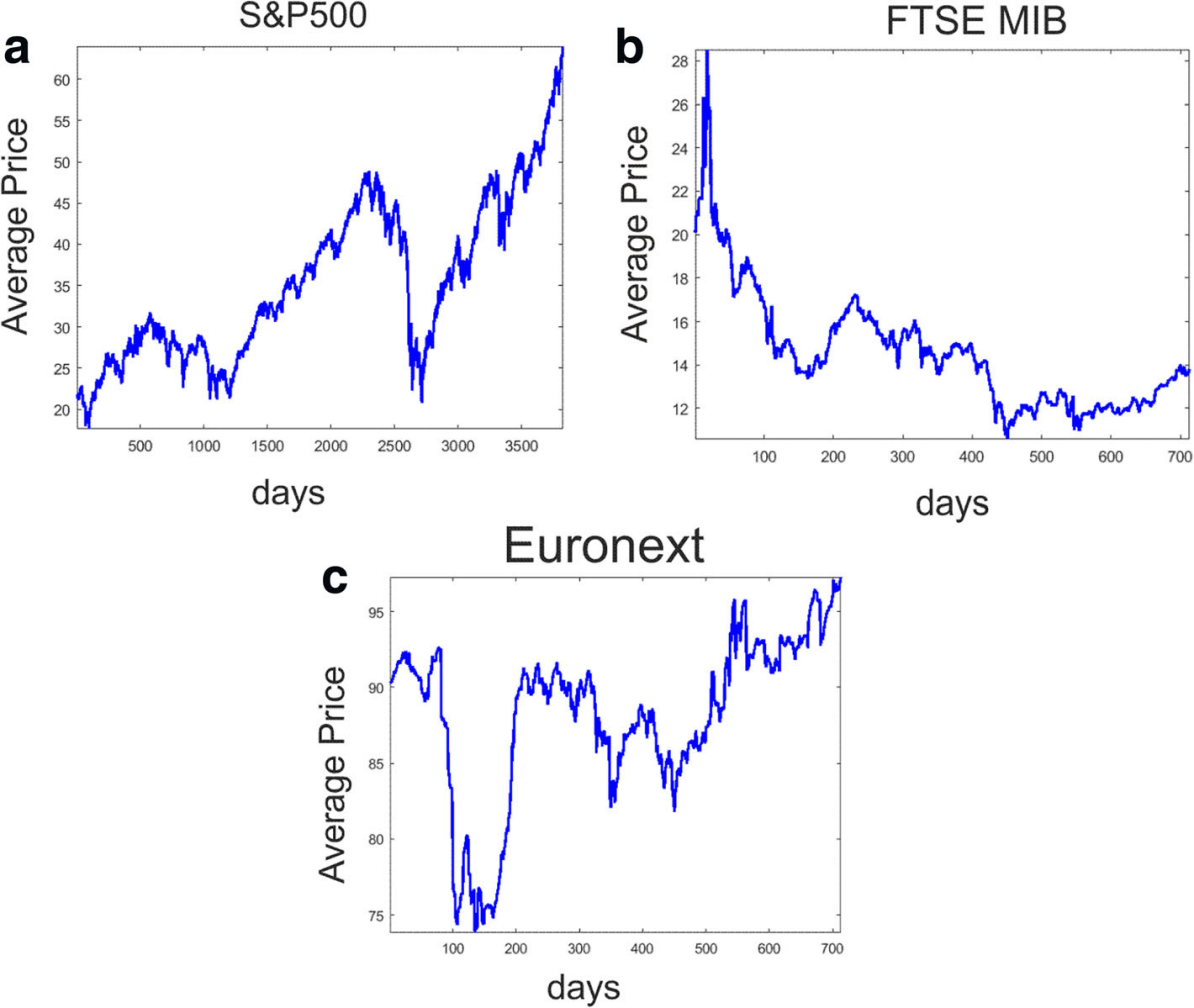
Average price dynamic for the 388 stock belonging to the first database (S&P500) during the period 1998–2013 (**a**) together with the average value of the 59 stocks of the second database (FTSE MIB) during the period 2014–2017 (**b**) and the average value of the 156 stocks of the third database (Euronext Paris) during the same years 2014–2017 (**c**). Starting from the boom phase of the dot-com bubble in 2000, Fig. (**a**) also shows the market decline of 2002 (near period 1000), the recovering period ended in 2007 with the sub-prime mortgage crisis together with the global financial crisis of the 2008–09 (near period 2700). In Fig. 2(**b**) the average price dynamic shows an abrupt increase in the first part of the sample, followed by a sudden decreasing phase and a more stationary behavior after 2015 (period 200). Similarly the average price dynamic of the third dataset in Fig. 2(**c**) shows a fall in 2015 (near period 120), at the time of parliamentary elections in Greece and the stationary phase after that

In order to give a rough overview of the markets dynamic during the time periods under analysis, Fig. 2(a) shows the average price of the basket of stocks composing the first database while Fig. 2(b) displays the average dynamic of the stocks employed in the second dataset and Fig. 2(c) illustrates the average price dynamic for the third database.

Besides the general increasing trend, Fig. 2(a) shows two broad recession periods, the collapse of the dot-com bubble occurred in 2002–03, and the global financial recession created by the bursting of the sub-prime mortgage bubble of 2008–09. Figure 2(b), instead, reports the average behavior of the stocks belonging to the FTSE MIB. The average price peaks at the beginning of the sample and then decreases sharply. The average price of the of 156 stocks composing the Euronext shows approximately the same fall near period 100 (see Fig. 2(c)). This phase indeed represents the time of parliamentary elections in Greece, a period that has negatively influenced almost all European stock exchanges. After this turmoil, the average price of the stocks belonging to these European stock exchanges reaches a stationary level.

**Fig. 3:**
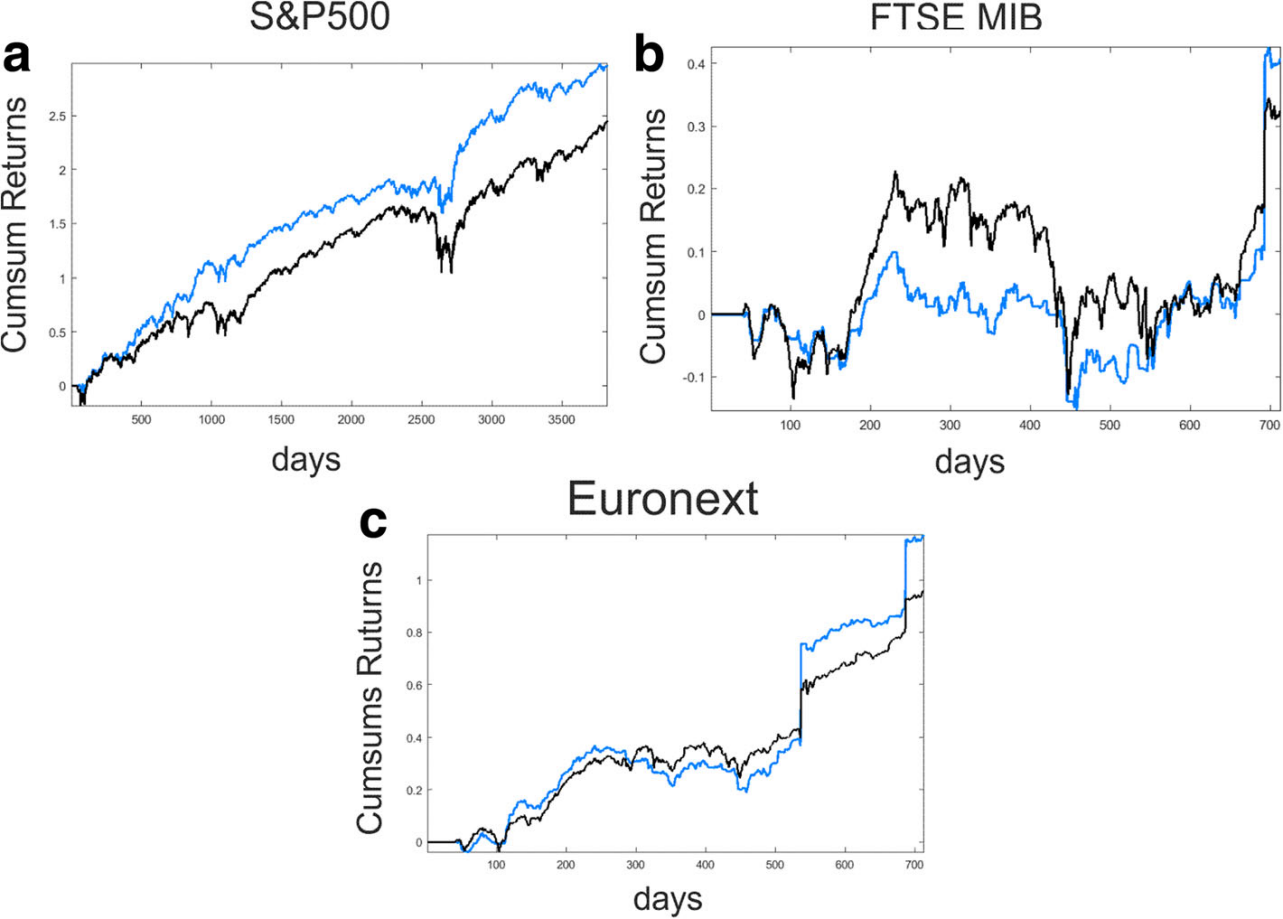
Cumulative sum of returns obtained with the signal-optimized portfolio strategies (*blue lines*) together with the cumulative performance of the equal weight investment strategy (*black lines*). Figure 3 **a** shows the performance of the investment strategy for the basket of stocks belonging to the S&P500. Panel **b** reports the results for the basket of stocks belonging to the FTSE MIB and panel **c** presents the cumulative sum of returns for the basket of stocks belonging to the Euronext Paris stock exchange

Figure 3(a) shows the cumulative sum of returns for the basket of stocks belonging to the S&P500 index obtained using the dynamic investment strategy based on signal-optimized portfolios (solid blue line) together with the cumulative performance obtained with an equally weighted portfolio (solid black line). The simulations are performed using the following parameters: ${n}_1=15$, $Z=25$, ${n}_2=7$, the exponential smoothing parameter is set to be equal to $0.2$ and the upper bound for the weights in the portfolio maximization problem constraint (23) equal to 0.1 meaning that the quantity invested in a particular stock cannot exceed the 10% of the total wealth. From Fig. 3(a) it is clear that the investment strategy based on signal-optimized portfolios performs better than the equally weighted strategy with a cumulative return at the end of the sample period of 296% against the 245%. This result is given by the fact that, even if in periods of business expansion the two strategies perform equal (see periods 1000–2200 or 3000–3500), the signal-optimized investment plan less suffers the drastic drop of stock prices during crisis phases. This behavior is evident near periods 2500–2800 that represent the 2007–08 financial crisis in which the equal weighted strategy loses approximately the 40% while losses of the signal-optimized portfolio are near the 18%.

Similarly Fig. 3(b) displays the cumulative sum of returns for the basket of stocks belonging to the FTSE MIB obtained using the signal-optimized portfolio strategy (solid blue line) together with the cumulative performance obtained with an equally weighted portfolio (solid black line). Also in this case the investment strategy based on signal-optimized portfolios performs better than the equally weighted strategy with a cumulative return at the end of the sample period of 41% against the 33%. The simulations are obtained using the same parameter values of the previous case. Also for these simulations it is clear that, even if in some periods of business expansion the equally weighted portfolio strategy performs better (see periods 100–200), the signal-optimized investment plan less suffers from decreasing phases as around period 450. Moreover after that period the cumulative returns obtained with this strategy are larger than the ones obtained with an equal portfolio investment plan.

Finally Fig. 3(c) presents the cumulative sum of returns for the basket of stocks belonging to the Euronext Paris stock exchange obtained with the signal-optimized portfolio strategy (solid blue line) together with the cumulative performance obtained with an equally weighted portfolio (solid black line). As previously illustrated, also in this case the equally weighted strategy is outdated by the signal-optimized portfolio investment plan. The simulations are performed using the same parameter values of the previous cases except for ${n}_2=9$.

Since the parameter space is huge a sensitivity analysis is needed for investigating the robustness of the method against the change in some parameter values. The sensitivity is obtained keeping fix all the parameters except ${\mathrm{n}}_1$ and ${\mathrm{n}}_2$ that take different values along the simulations. The aim is to investigate how the results change while parameters vary and to study how these changes influence the final returns.

**Fig. 4:**
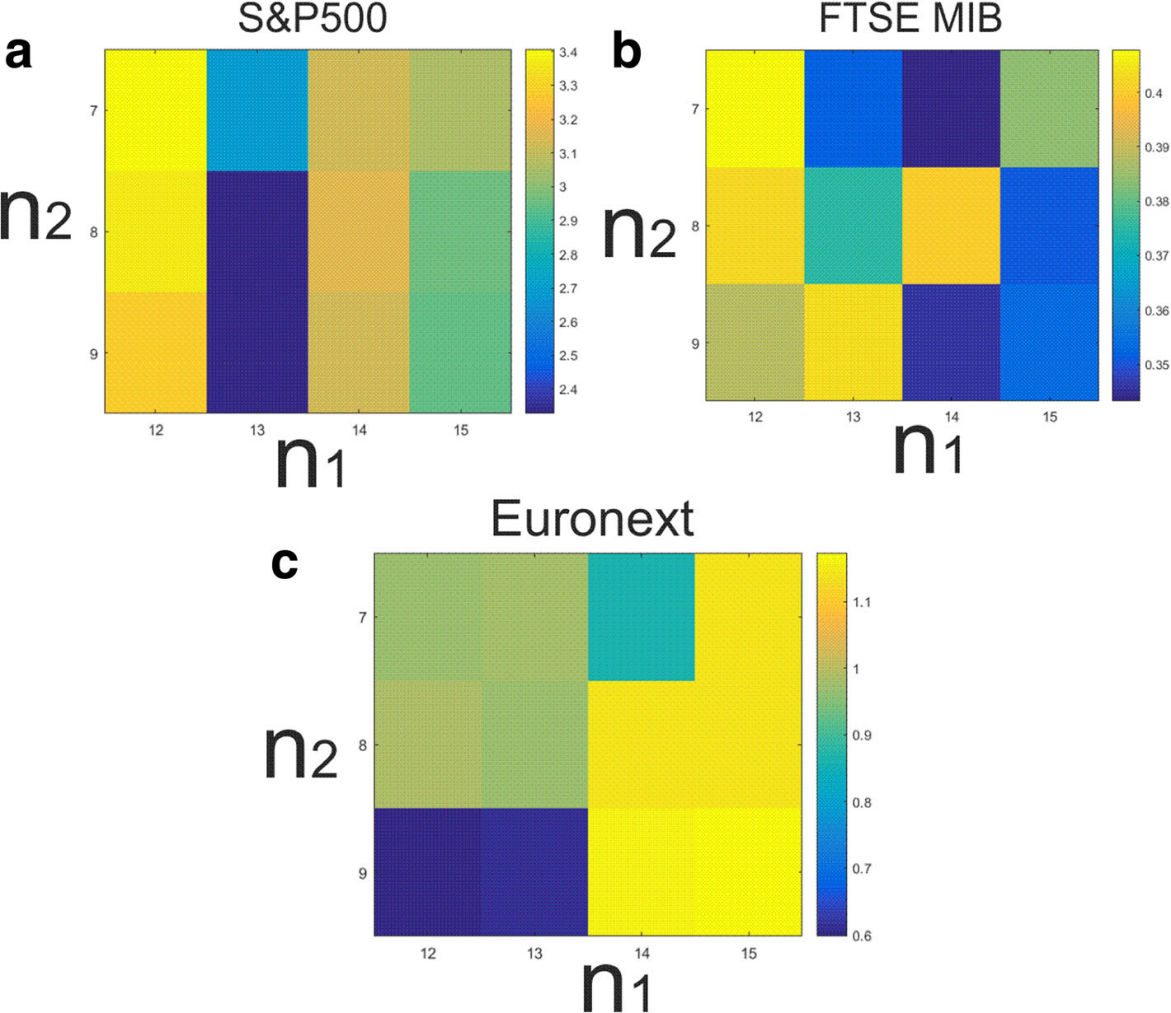
Cumulative returns obtained at the end of the time sample for different values of the parameters ${\mathrm{n}}_1$ and ${\mathrm{n}}_2$. Panel **a** represents the end-of-sample cumulative performance obtained in forecasting the dynamic of the 388 stocks of the first dataset (S&P500) . Panel **b** shows the end-of-sample cumulative performance obtained by the signal-optimized portfolio strategy in forecasting the dynamic of the 59 stocks of the second dataset (FTSE MIB). Panel **c** encompasses the cumulative returns obtained by the signal-optimized portfolio strategy in forecasting the dynamic of the 156 stocks of the third dataset (Euronext Paris stock exchange). The cumulative returns are positive for all parameter configurations suggesting some robustness of the proposed methodology against different setting of the parameters

Figure 4 presents, in each subplot, the sensitivity of the methodology in parameter variations by indicating the cumulative returns obtained at the end of the time sample. In particular, Fig. 4(a) regards the first database, Fig. 4(b) the second and Fig. 4(c) the third.

Results indicate that the cumulative returns are positive for all parameter configurations, this fact reinforces the idea that the proposed methodology is able to correctly predict most of the movements and that it is robust against different setting of the parameters.

Moreover, for most of the parameter configurations the signal-optimized portfolio strategy outperforms the equally weighted portfolio strategy. This is always true for the second dataset that represents stocks traded in the FTSE MIB (see Fig. 4(b)), while for some values of the parameters this is no longer true for the first and the third dataset, those values are ${n}_1=12:13$ and ${n}_2=9$ for the stocks traded in the S&P500 and ${n}_1=13$ and ${n}_2=8:9$ for the stocks traded in the Euronext Paris stock exchange.

## Discussion and concluding remarks

This work proposed a new dynamical approach to financial systems and stressed the systemic importance of empirical signs that can be used to extend the knowledge of financial markets and complex systems in general.

The results show that the complex approach to financial markets produces investment plans superior to simpler strategies. In this regard, I think that the superior performance of the proposed approach rests on two entangled pillars. The first pillar is the quantification of risk from an interconnectedness perspective that, per se, contains a new value-adding information.

The second pillar derives from the tensor decomposition technique presented that can effectively extract complex relationships from stock prices’ time series without aggregating the data. This method avoids the losses of crucial information about the system that can be observed only by holding the original time-varying nature of the records. This approach indeed might reduce estimation error issues in the process of portfolio formation, as one focuses on the evolution of the distances and not on its average value.

Furthermore, the findings obtained by the application of this methodology might have important consequences for the understanding of other financial systems like CDS markets and other derivative markets. Indeed, as pointed out by the recent financial crisis, financial systems are increasingly built on interdependencies and relationships that are difficult to predict and control. This feature is calling for more researches and applications of complex network techniques in economics.

Future research could investigate this conjecture by applying similar methodologies to the banking system so to predict future events of crisis and the cause of the crisis by investigating also the systemic importance of each financial institution within the system.

Predicting abrupt market down-turns, as a matter of fact, can facilitate the drafting of policies that can reduce the severity of financial crises, by decreasing the risk of global collapses of financial services by making economic networks more robust.
